# Application of Continuing Care in Patients with Mild Cerebral Hemorrhage

**Published:** 2019-10

**Authors:** Min ZHANG, Yang YU, Yujun CHEN, Guihong FAN

**Affiliations:** 1. Department of Neurosurgery, The First Affiliated Hospital of Qiqihar Medical University, Qiqihar, 161041, P.R. China; 2. Department of Neurology, The First Affiliated Hospital of Qiqihar Medical University, Qiqihar, 161041, P.R. China; 3. Department of Nursing, The First Affiliated Hospital of Qiqihar Medical University, Qiqihar, 161041, P.R. China

## Dear Editor-in-Chief

The onset of cerebral hemorrhage is relatively sudden, and the disease progresses rapidly, which can cause serious damage to the patient's health and increase the risk of death to a certain extent (1, 2). At this stage, for the treatment of cerebral hemorrhage, clinicians usually take surgical treatment (3), but after the surgical treatment, the patients lack of self-management awareness and skills, which is not good for prognosis.

Therefore, this study aimed to explore the application of continuous care in patients with mild cerebral hemorrhage.

The study subjects were 104 patients with mild cerebral hemorrhage who were discharged from our hospital after neurosurgery from January 2016 to December 2017 (determined by sample size calculation formula, α=0.05), the subjects were divided into observation group and control group using random number table method, 52 cases each. All patients were diagnosed according to the diagnosis of mild cerebral hemorrhage by CT or MRI. There were 30 males and 22 females in the observation group, and 33 males and 19 females in the control group. There were no significant differences in the general data of the two groups.

Informed consent was taken from the participants before the study and the study was approved by Ethics Committee of the hospital.

Patients in the control group were given routine nursing interventions according to the relevant nursing procedures of the department. On this basis, the observation group was given a medical-patient-management trinity continuous care model.

The details are as follows: 1) Preparation: A continuous nursing group was established. The team members collected data of patients and learned the understanding of the disease and psychological needs of patients and their families. 2) Procedure: According to the needs of patients and their families, the continuation plan was made for the patients to operate at home.

The members of the team took turns to guide the patients every month. 1) to help patients establish the confidence to actively treat diseases, to enable patients to relax, to alleviate adverse emotions; 2) to inform patients and family members to take medicine on time, and promptly correct the patients who do not take the medicine in time or refuse to take the medicine to ensure the continuity of the medication; 3) According to the current state of the patient (treatment and life), correctly assess the patient's self-management ability at home; 4) patients with mild cerebral hemorrhage need a reasonable diet and balanced nutrition, balanced meat and vegetable, quit smoking and drinking, go to bed early, avoid fatigue or get cold. The team manages the case data and nursing process of patients with mild cerebral hemorrhage, and promptly urges patients to follow the care plan. The neurological deficit score, motor function score, self-care ability and quality of life were compared before and after intervention in the two groups.

The data were analyzed by spss19.0 statistical software. The enumeration data were expressed by frequency and percentage, the comparison was performed by *x*^2^ test. The Measurement data were measured by (*x̄* ± *s*), and the *t* test was performed, *P*<0.05 for the difference was statistically significant.

After the intervention, both the deficit score and the motor function score of the observation group were better than the control group (*P*<0.05) (Table 1). After the intervention, the self-care ability of the observation group (295.98±87.45) was better than that of the control group (196.65±46.98) (*P*<0.05). After the intervention, the quality of life of the observation group (678.98±87.92) was better than that of the control group (498.09±43.09) (*P*<0.05).

**Table 1:** Comparison of neurological deficit scores and motor function scores between the two groups

***Group***	***Neurological deficit scores***		***Motor function scores***	
	***Before intervention***	***After intervention***	***Before intervention***	***After intervention***
Observation (n=52)	8.33±0.27	4.24±1.02	53.67±0.54	79.51±2.87
Control (n=52)	8.21±0.19	6.31±0.95	52.05±0.31	60.64±11.32
*t*	0.665	0.619	7.321	8.469
*P*	0.037	0.361	0.026	0.019

Early rehabilitation care could effectively promote the recovery of neurological function in patients (4). Besides, continuous care could improve the dysfunction of patients with ischemic stroke (5). The results of this study showed that the neurological deficit score, motor function score, self-care ability and quality of life of the experimental group were better than those of the control group. When patients with mild cerebral hemorrhage and their families are proficient in home care programs, the recovery of the patient's nerve function can be promoted; moreover, it helps to enhance the patient's sense of participation, improve their self-care ability, and improve their quality of life.
